# Targeting Key Inflammatory Mechanisms Underlying Heart Failure: A Comprehensive Review

**DOI:** 10.3390/ijms25010510

**Published:** 2023-12-29

**Authors:** Adamantia Papamichail, Christos Kourek, Alexandros Briasoulis, Andrew Xanthopoulos, Elias Tsougos, Dimitrios Farmakis, Ioannis Paraskevaidis

**Affiliations:** 1 Medical School of Athens, National and Kapodistrian University of Athens, 15772 Athens, Greece; adamantiapm@gmail.com (A.P.); chris.kourek.92@gmail.com (C.K.); abriasoulis@med.uoa.gr (A.B.); 2 Department of Cardiology, University Hospital of Larissa, 41110 Larissa, Greece; andrewvxanth@gmail.com; 3 Department of Cardiology, Hygeia Hospital, 15123 Athens, Greece; cardio@tsougos.gr; 4 Attikon University Hospital, Medical School of Athens, National and Kapodistrian University of Athens, 12462 Athens, Greece; farmakis.dimitrios@ucy.ac.cy

**Keywords:** inflammation, heart failure, immune system, signaling, cytokines, TNF-α

## Abstract

Inflammation is a major component of heart failure (HF), causing peripheral vasculopathy and cardiac remodeling. High levels of circulating inflammatory cytokines in HF patients have been well recognized. The hallmark of the inflammatory imbalance is the insufficient production of anti-inflammatory mediators, a condition that leads to dysregulated cytokine activity. The condition progresses because of the pathogenic consequences of the cytokine imbalance, including the impact of endothelial dysfunction and adrenergic responsiveness deterioration, and unfavorable inotropic effects on the myocardium. Hence, to develop possible anti-inflammatory treatment options that will enhance the outcomes of HF patients, it is essential to identify the potential pathophysiological mechanisms of inflammation in HF. Inflammatory mediators, such as cytokines, adhesion molecules, and acute-phase proteins, are elevated during this process, highlighting the complex association between inflammation and HF. Therefore, these inflammatory markers can be used in predicting prognosis of the syndrome. Various immune cells impact on myocardial remodeling and recovery. They lead to stimulation, release of alarmins and risk-related molecule patterns. Targeting key inflammatory mechanisms seems a quite promising therapy strategy in HF. Cytokine modulation is only one of several possible targets in the fight against inflammation, as the potential molecular targets for therapy in HF include immune activation, inflammation, oxidative stress, alterations in mitochondrial bioenergetics, and autophagy.

## 1. Introduction

A key contributor in the pathophysiology of heart failure (HF) is inflammation, a pathological underlying condition that is responsible for most abnormalities in the peripheral vascular function and cardiac remodeling. Patients with HF are reported to have elevated levels of inflammatory cytokines, including tumor necrosis factor-α (TNF-α), interleukin (IL)-1, and interleukin (IL)-6, appearing in both plasma and circulating leukocytes, as well as in the failing myocardium. The hallmark of the inflammatory imbalance is the insufficient production of anti-inflammatory mediators, a condition that leads to dysregulated cytokine activity. The condition progresses because of the pathogenic consequences of this cytokine imbalance, which include endothelial dysfunction, adrenergic responsiveness deterioration, and unfavorable inotropic effects on the myocardium [1,2,3]. Thus, understanding the intricate mechanisms of the inflammatory processes in HF and complex links within HF itself are crucial for the development of targeted therapeutic interventions that aim to improve patients’ outcomes [1]. This literature review provides valuable insights into the complex interplay between inflammatory processes and HF, highlighting the potential for innovative therapeutic approaches and the importance of precision medicine in disease management. Our goal is to provide important insights into the range of anti-inflammatory therapy alternatives, as well as the potential implications for novel therapeutic approaches. Though many studies have examined the role of inflammation in HF, our study is unique because it provides new knowledge and therapeutic options focused on specific mediators and pathways. Personalized anti-inflammatory medications based on the patient’s inflammation profile and the facilitation of resolution pharmacology that activates the reaching of pathways in HF are likely to advance future treatment. By concentrating attention on the singular aspects of cardiovascular disease, this study review can contribute to the further development of more effective and personalized HF treatment.

## 2. Mechanisms of Heart Failure and the Role of Inflammation

HF is based on a complicated interplay of inflammation, neurohormonal stimulation, and immune system alterations. Various important underlying processes have been proven. First of all, inflammation contributes significantly to the genesis of HF and HF with preserved ejection fraction (HFpEF) in particular [4,5]. In addition, it has been observed that an innate increased systematic or cardiac immune response runs throughout the entire inflammatory process on multiple HF subtypes. Inflammation in turn, which is often a chronic issue, is one of the major risk factors for cardiovascular disease (CVD) and the pro-inflammatory molecules in HF that cause a vicious cycle and disrupt the calcium homeostasis and mitochondrial function, thereby impacting myocardial contractility [5]. The pathogenesis and persistency of HF involves inflammatory cytokines, immune cells, and signaling pathways. Cytokine activation and its cross-talk with the adrenal axis, renin-angiotensin aldosterone system (RAAS), as well as the endothelin system, have been considered important elements in the development and exacerbation of HF. Cytokine response involving the participation of IL-1β and IL-6, TNFα, and Transforming Growth Factor-β (TGFβ) is known to be highly critical in the pathogenesis of HF and in regulating its inflammation-based process. As such, the modulation of cardiac inflammation has been identified as an attractive target for the treatment of HF, and has also been the focus of numerous clinical trials.

Secondly, HF progression is also strongly associated with the stimulation of neuro-hormonal processes, including the Sympathetic Nervous system (SNS), endocrine and immune systems. Insulin sensitivity disturbance, microvascular dysfunction and cardiac remodeling that cause aggravation of the disease may be associated with the activation of the above-mentioned processes. Thirdly, there is an increasing body of evidence that focuses on gut microbiota as well as their metabolites, which also plays a significant part in the physiopathology HF. Derived from gut microbiota metabolism imbalance, gut microbial-derived metabolites could be involved in cardiac dysfunction and inflammation. The heart may be connected with gut failure through heart–gut axis [6]. Moreover, Toll-like receptors (TLRs) and nod-like receptors (NLRs), as part of the innate immune response system, have also been suggested to be a new therapy target of HF. They act as upstream regulators of cytokine activation during the pathogenic inflammation associated with HF, and the role of these receptors in HF has been a recent area of intensive research with potential therapeutic applications [7].

In focusing on the underlying inflammatory processes of the disease, it is worth mentioning that inflammation occurs due to a complex interaction between pro-inflammatory cytokines, adhesion molecules, and oxidative stress. The disorder is characterized by a dysregulated immune response, which results in myocardial remodeling and, finally, cardiac dysfunction [1]. Patients with HF show high levels of pro-inflammatory cytokines, all of which are secreted in a continuous manner [8]: TNF-α, IL-1, -6, -8, -10, -10R, -33 and -18 [1], vascular endothelial growth factor (VEGF), high-sensitivity C-reactive protein (hs-CRP), brain natriuretic peptide (BNP), vascular cell adhesion molecule 1 (VCAM-1), C-C Motif Chemokine Ligand 2 (CCL2), monocyte chemoattractant protein (MCP)-1 [9], intercellular adhesion molecule-1 (ICAM-1) [10], myeloperoxidase (MPO), and inducible nitric oxide synthase (iNOS) [3]. They also activate various cells within the myocardium; which shows that they are not only confined to the immune system [8]. The severity, course of development and prognostic evaluation of the disease and its exacerbations are associated with them [3]. Additionally, higher levels of these inflammatory mediators injure the endothelium, resulting in decreased smooth muscle cell activity promoting vascular tone. However, other studies have reported higher levels of anti-inflammatory substances such as adiponectin and soluble Fas/soluble Fas ligand, which were associated with unfavorable outcomes [10,11,12].

It is pertinent to mention some specific molecules, which play a key role in the pathogenesis of HF. Firstly, IL-1 action in cardiovascular disease depends upon molecular pathways of IL-1R1-IL-1RAcP and activation of interleukin-1 receptor-associated kinases (IRAKs), as well as tumor necrosis factor receptor associated factor 6 (TRAF6) [13]. It causes the translation of these transcriptional messenger RNA factors p38 mitogen activated protein kinase (p38 MAPK) and nuclear factor kappa B (NF-kB) and, hence, its migration into the nucleus. Secondary messengers are molecules that are coded by hundreds of target genes and come at this point to create different kinds of signaling pathways [13]. Among the mechanisms linking IL-1 to impaired systolic function, IL-1 inhibits L-type calcium channels, uncouples the β-adrenergic receptor (β-AR) from the adenylyl cyclase (AC), and induces transcriptional and posttranslational changes in phospholamban and sarcoplasmic/endoplasmic reticulum calcium ATPase (SERCA) [13]. IL-1 also increases NOS expression, leading to increased nitric oxide (NO) activity. This further disrupts calcium and β-AR signaling and impairs mitochondrial function. These molecular mechanisms contribute to inflammation and cardiac dysfunction by promoting the production of proinflammatory cytokines and chemokines, and also by inducing contractile dysfunction in cardiomyocytes and reversible cardiomyopathy in animal models. Additionally, IL-1 has been shown to increase within hours in ischemic models of HF and is associated with the progressive nature of the cardiac dysfunction [13].

Other inflammatory markers such as MPO and CRP serve as indicators of cardiac stress and remodeling through their association with the pathophysiological processes of the disease. MPO, an enzyme released by activated neutrophils, has been linked to ventricular dysfunction and remodeling after myocardial infarction (MI), indicating its role as a marker of cardiac stress and remodeling [14,15]. On the other hand, CRP, an acute-phase reactant produced by the liver in response to inflammation, has been associated with the inhibition of pyruvate dehydrogenase activity and mitochondrial function of cardiomyocytes, further contributing to cardiac dysfunction and remodeling [14,15]. These mediators modulate the phenotype and function of myocardial cells. They have negative inotropic effects, inducing inflammatory activation in macrophages, stimulating microvascular inflammation and dysfunction, and promoting a matrix-degrading phenotype in fibroblasts. Additionally, they may exert chronic fibrogenic actions, leading to interstitial fibrosis, which may increase myocardial stiffness, further contributing to the pathogenesis of HFpEF [9]. Likewise, continuing the reference to interleukins, the continuous activation of oncostatin M (OSM), a cytokine of the IL-6 family, is implicated in cardiac remodeling and inflammatory cardiomyopathy [1]. It induces dedifferentiation of the cardiomyocytes, promoting the progression of HF in dilative cardiomyopathy [8].

Lastly, Bcl-2 interacting protein 3 (BNIP3) plays a significant role by mediating cell death caused by inflammatory cytokines. It has been found to be induced by TNF-α and NO molecules. It also mediates the detrimental effects of inflammatory agents, inducing IL-6 and resulting in the progression of HF. Additionally, its activation during stress or injury depletes endoplasmic reticulum Ca^2+^ and induces mitochondrial dysfunction and apoptosis [16]. The expression of BNIP3 is altered in human HF during inflammation response. Additionally, TNF-α has been found to up-regulate BNIP3 expression, further establishing the association between inflammatory mediators and BNIP3 [16]. Epigenetic regulation of BNIP3 has been shown to play a crucial role in the progression of various types of diseases, including the cardiovascular disorders. Hypomethylation of BNIP3 causes the up-regulation of the protein’s expression level and contributes to the development of coronary artery diseases (CAD) that profoundly provokes cardiac dysfunction [17].

Inflammatory markers contribute to cardiac dysfunction through various mechanisms. For instance, TNF-α has been shown to have negative inotropic effects on the adult mammalian heart, leading to impaired cardiac function and, thus, exacerbation of HF symptoms [14]. Additionally, IL-1 and IL-6, as well as CRP, have been associated with the inhibition of pyruvate dehydrogenase activity and mitochondrial function in cardiomyocytes, further contributing to cardiac dysfunction. Furthermore, MPO has been linked to ventricular dysfunction and remodeling after MI, while iNOS inhibition has been found to improve ventricular function and remodeling post-MI [14].

It is worth noticing the pivotal role that vascular endothelium has in the pathophysiology of HF by regulating vascular tone, inflammation, and thrombotic mechanisms. Altered endothelial function is a result of the reduced synthesis and release, along with increased degradation of NO, and elevated production of endothelin-1 (ET-1). Some measures that have a positive effect on the endothelial function are physical activity, along with the traditional and adjunctive treatments for HF [3]. An additional condition that contributes to the formation of inflammation in the context of all phases of HF is oxidative stress. It is generated by the induction of eNOS uncoupling, in addition to the regulation of apoptosis among endothelial cells [18].

Many other aspects of HF are triggered by the underlying inflammatory processes, and they have been observed in both HF with reduced ejection fraction (HFrEF) and HFpEF [10]. These include the left ventricular (LV) function, LV remodeling, cachexia, and hematopoiesis [10,12]. Thus, inflammatory biomarkers can serve as a prognostic clue to the appearance of all these alterations. In HFpEF, inflammation plays a crucial role, particularly in response to cardiac pressure overload (e.g., hypertension). In such conditions, inflammation is one of the earliest pathophysiologic findings; it appears with the elevation of endothelial adhesion molecules levels, with an increased production of inflammatory cytokines, and with the inflammatory infiltration (activated inflammatory cells) of the myocardium [19].

Cytokines aggravate chronic HF symptoms (e.g., hemodynamic imbalances), induce weight loss, and are directly cardiotoxic, resulting in a decline in myocardial tissue [17]. Chronic inflammation with elevated proinflammatory cytokine levels is considered to have a pathogenesis role in myocardial remodeling. This is established by impairing myocardial contraction, causing ventricular hypertrophy, and activating apoptosis mechanisms. The persistent inflammation involving increased levels of inflammatory cytokines plays a pathogenic role by influencing heart contractility, inducing hypertrophy, and promoting apoptosis, contributing to myocardial remodeling. The inflammatory mediators may, in giving significant prognostic information and increased levels of cytokines in HF patients, indicate major pathogenetic mechanisms [20]. It is shown that NF-κB activation and increased levels of CRP highlight the pathogenesis of immune perturbance in chronic HF. Comparatively, the development of HF is usually based on chronic inflammation processes, which occur, for example, among patients suffering from CAD. Necrosis is another type of cell death (the most frequent) associated with cellular pathology and HF, LV dysfunction, negative inotropic effects, changes in the cardiac metabolism, myocardial remodeling, and HF progression [16]. Activating signals, like heat shock proteins, High-mobility group box 1 (HMGB1), Adenosine Triphosphate (ATP), and ROS released by damaged myocytes and extracellular matrix work as initiators of inflammatory response. Furthermore, NO and Reactive Oxygen Species (ROS), mainly in the highly inflamed environment of ischemic heart disease, interact to cause increased cell injury, as well as maintain the inflammation. The latter ultimately leads to function failure of the myocardium [16].

There are certain comorbidities that contribute to persistent low-grade inflammation and exert deleterious effects on organ systems beyond the heart, such as skeletal muscle oxygen extraction during exercise, anemia, sarcopenia, sodium retention in the kidneys, and increased pulmonary pressures during exercise due to pulmonary vasoconstriction, all of which contribute to dyspnea and reduced exercise tolerance in HF [21]. It is equally interesting that evidence of viral infection in patients with HF and the associated immune response suggests a direct link between inflammation and HF. Additionally, chronic immune disorders like rheumatoid arthritis have been associated with an increased risk of HF, particularly the nonischemic type, indicating that chronic inflammation may directly lead to HF. In terms of immune response, while certain CD4+ regulatory T cells have been found to be cardioprotective, preventing the progression of HF, the balance of immune-mediated injury and repair remains poorly understood, especially in the chronic phase of myocardial injury [22]. If inflammation is a direct cause of HF, treatments targeting the immune response may be beneficial. However, if inflammation primarily serves as a marker of disease, immune modulatory treatment may not be effective, but targeting patients with documented inflammation to identify and treat unrecognized LV dysfunction could be beneficial [22].

## 3. Prognostic Biomarkers in Heart Failure

The use of anti-inflammatory medications in HF has been prompted by the recent finding that abnormally high levels of specific inflammatory biomarkers (e.g., NT-proBNP, CRP, pentraxin 3, and a combination of other serum markers) might predict future cardiovascular events. Prognostic biomarkers can be used for the evaluation and the guidance of any given treatment. However, challenges with their clinical surveillance have made a more focused strategy for identifying individuals who would benefit from specific approaches necessary [23]. CRP has been linked with characteristics of more severe stages of HF and has been found to be independently related to unfavorable outcomes, suggesting that it may be beneficial for determining if patients would benefit from treatment with statins [24]. Moreover, Anand et al. [25] showed that both CRP and TNF can independently predict morbidity and mortality. Additionally, Food and Drug Administration (FDA) recently approved two prognostic inflammatory biomarkers in HF, soluble ST2 and galectin-3, for the prediction of outcomes in HF patients. There is even greater growing interest in pentraxin-3 as a novel cytokine biomarker, further expanding the repertoire of inflammatory biomarkers used for prognostication [25]. In addition, the CD14+ + CD16+ monocyte population has been implicated as a potential inflammatory biomarker, with its levels correlating to the severity of HF, LV ejection fraction (LVEF), and pro-BNP levels. This suggests the potential of this monocyte population as an inflammatory biomarker [25]. IL-1 also plays a significant role in HF, as it is upregulated in HF and associated with a worse prognosis. IL-1 has been shown to induce contractile dysfunction in isolated cardiomyocytes and reversible cardiomyopathy in mice, as well as increase within hours in ischemic models of HF, and is associated with the progressive nature of cardiac dysfunction. Additionally, IL-1 blockade has been found to limit postinfarction ventricular remodeling, improve ventricular systolic and diastolic function, and increase survival in animal models. It has been also identified as a cardiodepressant factor in severe sepsis and has been linked to the presence of a circulating cardiodepressant factor of acute decompensated HF patients [13]. Hofmann and Frantz also pointed out that it is necessary to identify novel biomarkers, as well as novel imaging modalities to characterize the immunological status of each patient and to provide individualized treatment. If this happens, the suitability of patients with HF for the targeted immuno-modulation therapy will be determined more easily [26].

## 4. Anti-Inflammatory Treatment Strategy

Several anti-inflammatory medications have been studied, each with a different degree of effectiveness (Table 1) [12,13,19,22,23,27,28,29,30,31,32,33,34,35,36,37,38,39,40,41,42,43,44,45,46,47,48,49,50,51,52,53,54,55,56,57,58,59,60,61,62,63,64,65].

Many clinical trials were conducted, and most showed promising results in the limitation of inflammation, minimization of heart damage, as well as the prevention and outcomes of the disease itself [2,10,16,20,22,24,66,67,68]. All of these methods have provided accurate clinical evidence for the reduction of inflammation (Table 2) [51,69,70,71,72,73,74,75,76,77,78,79,80,81,82,83,84,85,86,87,88,89,90,91,92,93,94,95,96,97,98,99].

Inflammation can be inhibited or at least reduced by several methods. First, one major way is the restoration of intercellular glutathione metabolism. It may be also possible to neutralize the various inflammatory and proinflammatory cytokines involved in the generation of inflammation in HF context, such as IL-10, making it possible to reverse the various inflammatory pathways of HF [1,68].

Cytokine modulation is only one of several possible targets in the fight against inflammation. The potential molecular targets for therapy in HF include immune activation, inflammation, oxidative stress, alterations in mitochondrial bioenergetics, and autophagy [18,100]. Other approaches include the delivery of neutralizing IL-1β or IL-1 receptor antibodies to patients with abundant numbers of CCR2+ macrophages in the cardiac tissue; the inhibition of chemokines that potentiate inflammatory monocyte and T cell recruitment; and/or activation and targeting of IL-1β in atherosclerosis [15]. In two novel developments, the significance of the IL-10/TNF-α ratio and the influence of low-dose ionizing radiation on inflammation and oxidative stress in HF patients both offer future treatment opportunities [101]. Studies of both preclinical and clinical data revealed a decrease or reversal in endothelial dysfunction caused by the induction of NO synthesis and the administration of statins, antioxidants, l-arginine, and angiotensin-converting enzyme (ACE) inhibitors. Increasing data supports the application of intravenous immunoglobulin (IVIG) [1]. Moreover, the benefits of physical activity, cessation of smoking, growth hormone therapy, carnitine intervention, and angiotensin receptor blocker therapy in improving outcomes have been established in the literature [3].

Targeting inflammation in HF is a topic of an ongoing research, and is a preoccupation for some additional approaches that have recently emerged, in addition to the controlling of cytokines. New emerging targets of inflammation in HF include the administration of modern anti-inflammatory drugs, such as colchicine and methotrexate, which have been demonstrated to reduce inflammation and provide beneficial outcomes in HF patients. Furthermore, there are indications that altering the composition of gut microbiota may reduce inflammation and ameliorate symptoms. Additionally, novel treatments aimed at distinct pro-inflammatory cascades (e.g., NLRP3 inflammasomes) have been explored, with specific attention to their ability to reduce HF-induced inflammation. Other novel methods utilized include cancer drugs directed against PLK1, which have shown promise in the management of medical conditions like HF and cardiomyopathy. Moreover, a new specialized type of immunomodulatory cells has been prospectively mentioned in relation to a large cause of heart failure, namely inflammation. The proposed innovations are extremely promising and could lead to the design of new therapies that can be used to treat inflammation from a HF perspective.

Targeting OSM signaling may represent a meaningful therapeutic approach for the prevention of HF and the improvement of cardiac function. Inhibition of OSM signaling improves cardiac function in a mouse model of inflammatory cardiomyopathy. This is achieved by reducing cardiomyocyte remodeling and dedifferentiation, resulting in improved cardiac performance and increased survival. Pharmacological attenuation of long-lasting Ob signaling is a promising strategy to treat different types and stages of HF. Additionally, partial inhibition of OSM-receptor (Ob) activity improved cardiac function and reduced cardiac remodeling without optimization of the treatment regimen. This indicates potential therapeutic benefits in human patients. It may also aid the prevention of HF in aging societies [8].

One potential epigenetic-related therapeutic approach involves the up-regulation of BNIP3 in HF and epigenome-based drug discovery. This is because they seek to regulate the expression of this marker which in turn, might help to uncover novel therapeutic options [17]. Similarly, NOD-, LRR- and Pyrin Domain-Containing Protein 3 (NLRP3) inflammasome could also represent other targets of promising anti-inflammatory agents. It has also been proposed that CCL2 may be a potential target in diseases linked with cardiac injury, as well as in HF. Antagonism of the CCL2:CCR2 axis holds promise as a HF therapy, as it may contribute to the attenuation of inflammation-driven fibrosis [9,102].

In a State-of-the-Art Review [21], the existing evidence from clinical trials of anti-inflammatory treatment strategies in HF have been shown to be mostly unsuccessful, likely reflecting our poor understanding of the complex inflammatory networks within the syndrome of HF. The cornerstone of guideline-directed medical therapy for patients with chronic HFrEF involves the inhibition of the RAAS and sympathetic nervous system, as well as augmentation of favorable pathways with neprilysin inhibition. Biomarkers of inflammation are observed to decrease in patients receiving many such therapies. These effects are likely indirect, however, and related to improved HF, rather than direct anti-inflammatory effects [21,103,104].

Inflammation plays a significant role in HF, as evidenced by the association of acute and CHF with inflammatory cell activation. The use of anti-inflammatory approaches has shown efficacy in animal models and in small-scale clinical trials in humans. These anti-inflammatory effects are thought to be mediated, at least in part, by the induction of regulatory T cells (Tregs) and the promotion of acetylation of the FoxP3 transcription factor, which is a master regulator of Treg differentiation. The use of anti-inflammatory drugs in conjunction with standard HF therapies, such as ACE inhibitors and β-blockers, presents an opportunity for synergistic treatment strategies. While the efficacy of anti-inflammatory agents in HF is promising, further research is needed to elucidate the mechanisms of action and to determine the optimal dosing and safety profiles for these agents in the context of HF [105].

Hofmann and Frantz [26], included an outline of several randomized controlled trials that evaluated the clinical outcomes of the administration of immunomodulating, or anti-inflammatory agents given in chronic HF, in their study: the improvement of many functional parameters, including New York Heart Association (NYHA) class, LVEF, possibility of hospitalization, cardiac remodeling, and mortality rates was obvious in most of them [26].

Inflammation targeting therapies in HF clinical trials have not been promising and there are multiple explanations why. For example, CORONA (Controlled Rosuvastatin Multinational Trial in Heart Failures) demonstrated a decrease in high sensitivity CRP, without mortality reduction in older patients with HF [106]. The same was found in a GISSI-HF trial that investigated if rosuvastatin could decrease mortality and hospitalization of HF [107]. Moreover, some trials with anti-TNF agents were abandoned on the grounds of futility and harm to the intervention group. These were also like other placebo-controlled IVIG and immunomodulation of cytokine trials. Additionally, corticosteroids have shown improvement in LVEF in studies and metanalysis of patients with acute myocarditis [22].

It is necessary to highlight the fact that, despite the promising results that the available anti-inflammatory medications have shown, further research is essential to identify the target molecules of the treatment with a greater specificity [1,68]. Moreover, mortality and morbidity rates are still high, despite the use of conventional treatments targeting neurohumoral activation, emphasizing the need for additional therapeutic options. Similarly, while it is well-established that inflammation has a key role in the pathogenesis and progression of HF, targeting inflammation with specific therapeutic agents may not be universally effective [108]. Precise patient selection is crucial when considering additional therapies, as different subgroups of patients may exhibit varying levels and types of the inflammatory cascade. For instance, inflammatory activation may differ in HF occurring in the early stages after acute MI compared to chronic HF, and different types of HF (e.g., diabetic, ischemic, hypertensive, viral, and idiopathic cardiomyopathy) may require the target of different inflammatory pathways [16].

## 5. Discussion

The central cytokine in the pathogenesis of HF is TNF-α, yet its modulation is another potential therapeutic approach. Preliminary reports suggested that TNF-α inhibition with recombinant chimeric soluble TNF receptor (TNF-R) type 2 (etanercept) may have beneficial effects on cardiac remodeling [16]. However, studies with a large number of participants that examine the impact of etanercept on morbidly and mortality have shown conflicting results in HF [16].

It has been suggested that inflammation is a result of the impairment of cardiomyocyte homeostasis due to imbalanced activity between protein synthesis-degradation and organelle capacity. Moreover, apoptogenic proteins cannot be eliminated and damaged mitochondria are not activated and well-functioning. As a result, the cardiomyocyte death along with extra-cellular cardiac matrix dysregulation, lead to myocardial cellular dysfunction and ultimately to HF [109].

One therapeutical approach is the use of statins, which have anti-inflammatory properties and can reduce pro-inflammatory cytokines (such as IL-1, IL-6, and TNF-α from macrophages), inhibit cell adhesion molecules, and augment NO production. However, randomized studies of statins in patients with HF have not supported their use in this population unless indicated by the presence of CAD or dyslipidemia [16]. The Scandinavian simvastatin survival study (4S) demonstrated fewer instances of new-onset HF after simvastatin treatment, suggesting potential benefits of statin treatment in HF. However, further prospective data are needed to examine their effects and to determine the optimal doses needed to achieve the optimal anti-inflammatory effect [17]. Other studies have suggested that they may have immunomodulatory effects and could potentially influence the inflammatory cytokine cascade [20].

Although anti-inflammatory therapy has great potential, developing ideal immunomodulatory therapies has proven to be rather challenging. Extensive studies involving TNF-α antagonism that use etanercept as well as infliximab in a group of large-scale clinical trials have not demonstrated any benefit for chronic HF patient populations, further exemplifying the intricacy behind the inflammatory mechanism involved. In addition, the multiple causality for chronic HF and the complex character of the immune reaction are both problems for attempts to find appropriate treatment [1,68]. The complex interplay between inflammatory mediators and the cardiac extracellular matrix underscores the need for continued research to develop new treatment strategies targeting inflammatory and immunopathogenic mechanisms in HF [24]. The importance of well-designed clinical trials and the identification of patient subpopulations that may benefit from targeted cytokine or chemokine inhibition is also underscored.

Moreover, the challenges faced in translating experimental findings on cytokine inhibition into clinically applicable treatment are multifaceted. Firstly, documentation of clinical benefits in human HF populations requires well-designed and expensive clinical trials with long follow-up, leading to a slow pace of testing candidate agents. Secondly, animal models are suboptimal for predicting therapeutic efficacy due to their inability to recapitulate the pathophysiologic heterogeneity of human HF. Thus, a major priority in clinical research is the pathophysiologic stratification of human patient populations and the identification of patient subsets with excessive or dysregulated inflammatory responses. Thirdly, the need for chronic administration of cytokine or chemokine inhibitors to delay progression of HF is a major concern, as it poses potential risks and challenges [102].

Neutrophil-to-lymphocyte ratio (NLR) has been investigated as a recent inflammatory index and a prognostic tool in HF during the last few years. It is a simple prognostic tool for risk stratification and prioritizing high risk patients in clinical settings, especially in resource-limited nations [110]. In a recent meta-analysis [110], it was found that mean NLR in HF patients was 4.38 (95% CI: 4.02–4.73). Each unit increase in this biomarker has been associated with an increased mortality risk of 1.12 (95% CI: 1.02–1.23, *p*  =  0.013) times, and this risk was higher among patients with higher NLR values than proposed cut-offs (HR: 1.77, 95% CI: 1.27–2.46, *p*  =  0.001); being in a higher NLR tertile was associated with increased death likelihood (T2 vs. T1: HR: 1.56, 95% CI: 1.21–2.00, *p*  =  0.001, T3 vs. T1: HR: 2.49, 95% CI: 1.85–3.35, *p*  <  0.001) [110]; and NLR could be considered as a biomarker that mirrors the balance between acute and chronic inflammation and adaptive immunity, as well as a robust prognostic marker of disease severity and predictor of mortality in several diseases, including sepsis, pneumonia, COVID-19, cancer and cardiovascular diseases [111].

## 6. Conclusions

Targeting the key inflammatory mechanisms that underlie HF seems quite a promising approach for a therapeutic strategy of HF. Although there are currently anti-inflammatory therapeutic approaches, knowledge on this field still remains limited. The inflammation process in HF has been associated with an unbalanced level of cardiomyocyte homeostasis, resulting in cell death and the malfunctioning of the extracellular cardiac matrix. Of the several cytokines associated with HF, TNF-α, which showed modulative activity on cardiac remodeling, has been identified as a central one. However, investigations of etanercept as a TNF-α inhibitor in HF have proven to be limited in number and controversial. Several studies have also addressed statins with anti-inflammatory characteristics, which are meant to reduce pro-inflammatory cytokines. However, randomized control trials of their efficacy have not supported their use in HF, unless there is concomitant CAD or dyslipidemia. In addition, the challenges involved in developing effective immunomodulatory therapies include the complex nature of the immune response and the difficulty of translating scientific knowledge into effective clinical treatment. These reiterate the requirement for proper, well-designed, expensive clinical trials that have prolonged follow-up, that acknowledge the failure of animal models to predict drug efficacy, and safety concerns related to the long-term application of cytokine inhibitors and chemokines. Randomized controlled trials should be undertaken to gain an enhanced understanding of possible sources of inflammation, as well as the possibility of creating new anti-inflammatory strategies. More scientific studies need to come up with new disease therapies that to block inflammatory/immunopathogenic factors implicated in HF development, and also identify patient subgroups who would respond to specific chemokined or cytokined inhibition strategies.

## Figures and Tables

**Table 1:** An overview of the anti-inflammatory therapeutic options in HF. Each agent targets a specific inflammatory process, offering potential opportunities for a novel therapeutic intervention.

Anti-Inflammatory Therapy	Mechanism of Action	Clinical Evidence/Application
TNF-α Inhibitors (e.g., Infliximab, Etanercept, Adalimumab) [27,28,29]	Inhibits TNF-α signaling, reduces inflammation, and improves cardiac function.	Clinical trials have shown mixed results, with some studies suggesting potential benefits. Risk of infections; limited long-term data. May be prescribed in patients with inflammatory cardiomyopathies secondary to viral infection or systemic diseases. May have beneficial effects on cardiac performance, decrease inflammation and improve outcomes. Soluble TNF receptors have been investigated as potential immunomodulators. They may act as decoy receptors, binding to TNF-α and reducing its inflammatory effects. **RENEWAL**: Recover Renaissance trial showed equivocal results, with mainly beneficial or neutral outcomes.
Statins [30]	Anti-inflammatory actions in addition to lipid-lowering effects.	Variable impact on inflammatory biomarkers and clinical outcomes. Exert anti-inflammatory properties by reducing pro-inflammatory and inflammatory cytokines (e.g., IL-1, IL-6, TNF-α) and by improving clinical outcomes. Beyond their lipid-lowering effects, they possess anti-inflammatory properties and their potential role in modulating the inflammatory response has been investigated. Short-term atorvastatin treatment has been found to improve endothelial function by reducing the expression of proinflammatory cytokines and adhesion molecules. Adverse effects, statin intolerance.
Corticosteroids [31,32]	Suppression of inflammatory response. Inhibition of the production of inflammatory cytokines and reduction of inflammation.	Limited data in HF. Their anti-inflammatory effects in the context of acute HF exacerbations and post-operative inflammatory responses following cardiac surgery have been explored. Their potential role in mitigating inflammation and improving HF outcomes has been a subject of interest.
Interleukin-1 (IL-1) Blockade (e.g., Anakinra, Rilonacept, Canakinumab) [33,34,35]	Inhibits IL-1 signaling, reduces inflammation, ameliorates ventricular remodeling, and improves exercise capacity.	Preclinical studies and pilot clinical trials support the beneficial effects of IL-1 blockade in reducing systemic inflammation, potentially enhancing cardiac remodeling, and improving outcomes. A CANTOS trial demonstrated a 15% relative risk reduction in the primary composite endpoint of death, nonfatal stroke, and nonfatal MI. Experimental evidence has shown that IL-1 blockade may lead to a nearly 90% decrease in plasma concentration of IL-1β, suggesting a potential regulatory role of IL-1 activity in HF. IL-1 has been implicated in ischemia-induced systolic dysfunction, and IL-1 blockade has shown promising results in preserving systolic function and contractile reserve in animal models. Their efficacy and safety in patients with HFpEF is currently being investigated. Canakinumab has demonstrated improved left ventricular remodeling and reduction in cardiomyocyte apoptosis in mice after AMI. Anakinra has been shown to reduce cardiac apoptosis and improve cardiac function in rodent models of AMI. It has beneficial anti-inflammatory and anti-remodeling effects. In patients with STEMI, its subcutaneous administration led to reduced levels of IL-6 and CRP.
IL-6 blockade (e.g., Tocilizumab) [36,37,38,39]	Inhibition of IL-6, a proinflammatory cytokine.	Its potential to improve LV remodeling in mice has been investigated. Ongoing research and clinical trials are evaluating the efficacy of IL-6 blockade in HF.
Colchicine [40,41,42]	Inhibits microtubule polymerization, reduces inflammasome activation, and decreases cytokine production.	Clinical trials have demonstrated reduced cardiovascular events and improved outcomes in patients with CAD and MI.
Methotrexate [43,44]	Inhibits dihydrofolate reductase, reduces cytokine production, and modulates immune responses.	Limited clinical evidence, but studies of rheumatoid arthritis have shown potential cardiovascular benefits (CIRT trial).
Endothelin receptor antagonists (e.g., bosentan, macitentan) [45,46,47]	Inhibition of the effects of endothelin on the myocardium and vasculature.	Used to block the effects of endothelin-mediated myocardial and vascular remodeling in HF. They have been shown to improve symptoms and exercise capacity.
Antioxidants (e.g., vitamins C and E, coenzyme Q10) [48,49,50,51]	Scavenge ROS and prevent oxidative damage to the myocardium, potentially reducing oxidative stress and its impact on MI.	Their ability to mitigate the adverse effects of oxidative stress in HF has been studied. Studies suggest that oxidative stress can increase cytokine levels, representing a potential vicious cycle in HF, meaning antioxidative therapy could therefore be of interest.
NF-κB inhibition [52]	Suppression of NF-κB, a transcription factor involved in the expression of proinflammatory cytokines and adhesion molecules.	Pimobendan showed improvement in the management of HF in the EPOCH study.
NSAIDs	Inhibition of the activity of COX enzymes, reduction of the production of prostaglandins and other inflammatory mediators.	NA
MMP-2 inhibitors (e.g., PG-116800) [53]	NA	Their potential to prevent ventricular remodeling after MI has been investigated.
MMP-9 deletion [54]	NA	It has been shown to attenuate LV enlargement and collagen accumulation after experimental MI.
Soluble TNF-α receptor 1 gene transfer [55]	NA	It has been found to improve cardiac function and reduce infarct size after MI in rats.

Abbreviations. TNF-α: Tumor Necrosis Factor alpha; HF: Heart Failure; IL: Interleukin; ROS: Reactive Oxygen Species; MI: Myocardial Infarction; AMI: Acute Myocardial Infarction; NF-κB: Nuclear factor kappa B; NSAIDs: Nonsteroidal Anti-Inflammatory Drugs; COX: cyclooxygenase; LV: left ventricular; EPOCH Study: Effects of Pimobendan On Chronic Heart failure; MMP-2: Matrix metallopeptidase 2; MMP-9: Matrix Metallopeptidase 9; NA: Non-Applicable.

**Table 2:** Potential anti-inflammatory drugs and targets whose potential in addressing inflammation in heart failure is being investigated.

Anti-Inflammatory Strategy	Clinical Clues
Pentraxin-3 [69]	Emerging novel therapeutic target.
Mannose-binding lectin [70]	Emerging novel therapeutic target.
PI3Kγ [71,72]	Emerging novel therapeutic target.
Matricellular proteins [73]	Emerging novel therapeutic target.
RAAS antagonists [74,75,76]	They have been found to decrease the levels of cytokines in small clinical studies. There are potential benefits of ARB in improving outcomes exercise performance, hemodynamics, inflammation markers, and cardiac function in patients with CHF.
Beta-blockers [77,78]	They decrease the levels of proinflammatory cytokines in small clinical studies.
Antidepressants (e.g., SSRIs, SNRIs, TCA) [79]	They reduce the levels of inflammatory cytokines (e.g., TNF-α, CRP) in patients with HF and depression. The level of reduction in inflammatory agents depends on the kind of antidepressant agent administered, with patients treated with SNRIs or TCA showing lower levels of TNF-α and CRP than those treated with SSRIs.
Other anti-inflammatory agents	They target the complement cascade, cytokine system, and immune cells. They aim to attenuate inflammatory response and its detrimental effects on cardiac function.
Antioxidant agents [51,80,81,82]	A small double-blind randomized controlled trial investigating the use of vitamin E in HF patients did not demonstrate significant improvements. Other potential antioxidant approaches include targeting specific sources of oxidative stress and using medications with antioxidant effects, such as hydralazine and isosorbide dinitrate. There are potential benefits of carnitine treatment in improving outcomes exercise performance, hemodynamics, inflammation markers, and cardiac function in patients with CHF.
Physical Training [83,84,85]	Modulation of proinflammatory cytokines and the soluble Fas/soluble Fas ligand system. There are potential benefits in improving outcomes exercise performance, hemodynamics, inflammation markers, and cardiac function in patients with CHF.
Adiponectin [86]	Elevated levels associated with adverse clinical outcomes.
Soluble Fas/Soluble Fas Ligand [87,88]	Elevated levels associated with adverse clinical outcomes.
Omega-3 Fatty Acids [89,90,91]	Potential anti-inflammatory effects.
Growth Hormone administration [92,93]	There are potential benefits in improving outcomes exercise performance, hemodynamics, inflammation markers, and cardiac function in patients with CHF.
Immunomodulating Agents (e.g., IVIG, immunoadsorption, thalidomide, re-administration of autologous blood exposed ex vivo to oxidative stress, chemokine antagonists) [94,95]	Their potential to modulate the cytokine network and restore an inflammatory imbalance has been studied. Although they have demonstrated promising results in smaller studies, there is a need for confirmation in larger trials that feature hospitalizations and mortality as endpoints. Thalidomide has been studied as a potential treatment option. It has been shown to have anti-inflammatory effects and may modulate the cytokine network in HF patients. IVIG contains anti-inflammatory antibodies and its effects on immune activation and inflammation in HF patients have been studied. By reducing circulating inflammatory mediators, immunoadsorption could potentially modulate the immune response and improve cardiac function. They may target specific chemokines involved in the inflammatory response and myocardial remodeling in HF.
NF-κB inhibitors [96]	It has been shown to be upregulated in human myocardial tissue and is associated with the expression of proinflammatory cytokines, adhesion molecules, and effector enzymes.
Pentoxifylline, (xanthine-derived agent) [97,98]	It demonstrated beneficial effects in CHF in several clinical trials by inhibiting the production of TNF-α and being associated with improvements in HF symptoms and LV function.
HDAC inhibitors [99]	They have been found to demonstrate potent anti-inflammatory actions and have shown promise in blocking adverse cardiac remodeling in animal models. They target inflammatory cascades by affecting multiple cell types and diverse pathological mechanisms. They have been shown to block adverse cardiac remodeling characterized by myocyte hypertrophy, myocyte death, and fibrosis, ultimately leading to impaired cardiac function and HF.

Abbreviations: PI3Kγ: Phosphatidylinositide 3-Κinase-γ; RAAS: Renin-Angiotensin-Aldosterone System; ARB: Angiotensin Receptor Blockers; CHF: Chronic Heart Failure; SSRIs: Selective Serotonin Reuptake Inhibitors; SNRIs: Serotonin and Norepinephrine Reuptake Inhibitors; TCA: Tricyclic Antidepressants; CRP: C-Reactive-Protein, HF: Heart Failure; NF-κB: Nuclear factor kappa B; IVIG: Intravenous Immunoglobulin; HDAC: Histone deacetylase inhibitors.

## Data Availability

Not applicable.

## Abbreviations

AC	Adenylyl Cyclase
ACE	Angiotensin Converting Enzyme
ACEi	Angiotensin Converting Enzyme Inhibitors
AMI	Acute Myocardial Infarction
ARBs	Angiotensin Receptor Blockers
ATP	Adenosine Triphosphate
BNIP3	Bcl-2 Interacting Protein 3
BNP	Brain Natriuretic Peptide
Ca^2+^	Calcium Cation
CAD	Coronary Artery Disease
CCL2	C-C Motif Chemokine Ligand-2
CCR2	C-C chemokine Receptor type 2
CD16+	Cluster of Differentiation-16+
CD4+	Cluster of Differentiation-4+
CHF	Chronic Heart Failure
CORONA	Controlled Rosuvastatin Multinational Trial in Heart Failures
COX	Cyclooxygenase
CRP	C-Reactive Protein
CVD	Cardiovascular Disease
EF	Ejection Fraction
eNOS	endothelial Nitric Oxide Synthase
EPOCH Study	Effects of Pimobendan On Chronic Heart failure
ET-1	Endothelin-1
FDA	Food and Drug Administration
FoxP3	Forkhead Box P3
HDAC	Histone deacetylase inhibitors
HF	Heart Failure
HFpEF	Heart Failure with preserved Ejection Fraction
HFrEF	Heart Failure with reduced Ejection Fraction
HMGB1	High Mobility Group Box-1
hs-CRP	high-sensitivity C-Reactive Protein
ICAM-1	Intercellular Adhesion Molecule-1
IL-1	Interleukin-1
IL-10	Interleukin-10
IL-16	Interleukin-6
IL-1β	Interleukin-1β
IL-1R1	Interleukin-1 Receptor type 1
IL-1RAcP	Interleukin-1 Receptor Accessory Protein
iNOS	inducible Nitric Oxide Synthase
IRAKs	Interleukin-1 Receptor-Associated Kinases
IVIG	Intravenous Immunoglobulin
LV	Left Ventricular
LVEF	Left Ventricular Ejection Fraction
MCP-1	Monocyte Chemoattractant Protein-1
MI	Myocardial Infarction
MMP-2	Matrix metallopeptidase 2
MMP-9	Matrix Metallopeptidase 9
MPO	Myeloperoxidase
NA	Non-Applicable
NF-kB	Nuclear Factor kappa Beta
NLRP3	NOD-LRR-and Pyrin Domain Containing Protein-3
NLRs	Nod-like Receptors
NO	Nitric Oxide
NOS	Nitric Oxide Synthase
NSAIDs	Nonsteroidal Anti-Inflammatory Drugs
NT-proBNP	N-Terminal pro B-type Natriuretic Peptide
NYHA	New York Heart Association
OSM	Oncostatin-M
p38 MAPK	p38 Mitogen Activated Protein Kinase
PI3Kγ	Phosphatidylinositide 3-Κinase-γ
PLK1	Polo-Like Kinake-1
pro-BNP	pro B-type Natriuretic Peptide
RAAS	Renin-Angiotensin-Aldosterone System
RNA	Ribonucleic Acid
ROS	Reactive Oxygen Species
SERCA	Sarcoplasmic/Endoplasmic Reticulum Calcium ATPase
SNRIs	Serotonin and Norepinephrine Reuptake Inhibitors
SNS	Sympathetic Nervous System
SSRIs	Selective Serotonin Reuptake Inhibitors
ST2	Suppression of Tumorigenicity-2 protein
TCA	Tricyclic Antidepressants
TGFβ	Transforming Growth Factor beta
TLRs	Toll-like Receptors
TNF	Tumor Necrosis Factor
TNF-R	TNF-a inhibition with recombinant chimeric soluble TNF Receptor
TNF-α	Tumor Necrosis Factor-alpha
TRAF6	Tumor Necrosis Factor Receptor Associated Factor-6
Tregs	regulatory T cells
VCAM-1	Vascular Cell Adhesion Molecule-1
VEGF	Vascular Endothelial Growth Factor
β-AR	β-Adrenergic Receptor
